# Detection of DKD stage 1 and treatment are essential

**DOI:** 10.1080/0886022X.2018.1462208

**Published:** 2018-04-18

**Authors:** Narisa Futrakul, Prasit Futrakul

**Affiliations:** a Department of Physiology, King Chulalongkorn Memorial Hospital, Chulalongkorn University, Bangkok, Thailand;; b Academy of Science, The Royal Institute of Thailand and Bhumirajanagarindra Kidney Institute, Bangkok, Thailand

Sir,

It has been a general acceptance that early stage of diabetic kidney disease (DKD stage 1) has been underrecognized, and therapeutic prevention of the diabetic kidney disease at this stage has never happened. The significance and importance of the physiologically defensive mechanism and vascular homeostasis in the early stage of diabetic kidney disease has recently been identified [1]. Explanation for the underrecognition of DKD stage 1 is due to the overestimation of glomerular filtration rate resulting in the high flow of the blood through glomerular filtration (hyperfiltration state) at the expense of peritubular capillary blood flow which is reversely reduced [2]. Therefore, the diagnosis of DKD stage 1 cannot rely on the standard assessment of GFR or creatinine clearance, which is usually high or normal. To overcome the above handicap, an alternative approach has addressed to other functional, but more sensitive assessments such as the biomarkers that reflect tubulointerstitial disease, or biomarker of renal microvascular disease in particular peritubular capillary flow reduction that correlates with the severity of diabetic kidney disease. With respect to the former, fractional excretion of magnesium (FE Mg) – a biomarker that reflects tubulointerstitial fibrosis, has been demonstrated to be sensitive marker that is able to detect early stage of tubulointerstitial fibrosis [3], and inversely correlate with the degree of peritubular capillary flow reduction [4]. With respect to the latter, microvascular dysfunction such as peritubular capillary flow reduction, an increased arteriolar resistance and endostatin has been demonstrated in early stage of diabetic nephropathy associated with normoalbuminuria, normotension, normal creatinine clearance [2,5]. Another vascular biomarkers reflecting macrovascular disease such as abnormal values of endothelin-1, angioconverting enzyme (ACE) endostatin, cardio ankle vascular index have already been revealed in DKD stage 1 [6].

The preceding information renders support that DKD stage 1 can be recognized in the early stage with alternative diagnostic markers than creatinine clearance. The most important issue of early recognizing DKD stage 1 is that it is the golden period for implementing the therapeutic strategy to restore the renal microvascular disease as well as renal function, inasmuch as the mechanism of vascular repair and homeostasis is still adequately functioning in this stage. Treatment at this stage has been vulnerable to correct the renal ischemia, and restore the renal function, and effectively prevent the progression toward end-stage renal disease [7].
